# ^1^H, ^13^C, and ^15^N backbone chemical shift assignments of the apo and the ADP-ribose bound forms of the macrodomain of SARS-CoV-2 non-structural protein 3b

**DOI:** 10.1007/s12104-020-09973-4

**Published:** 2020-08-14

**Authors:** F. Cantini, L. Banci, N. Altincekic, J. K. Bains, K. Dhamotharan, C. Fuks, B. Fürtig, S. L. Gande, B. Hargittay, M. Hengesbach, M. T. Hutchison, S. M. Korn, N. Kubatova, F. Kutz, V. Linhard, F. Löhr, N. Meiser, D. J. Pyper, N. S. Qureshi, C. Richter, K. Saxena, A. Schlundt, H. Schwalbe, S. Sreeramulu, J.-N. Tants, A. Wacker, J. E. Weigand, J. Wöhnert, A. C. Tsika, N. K. Fourkiotis, G. A. Spyroulias

**Affiliations:** 1 Magnetic Resonance Center – CERM, University of Florence, Via Luigi Sacconi 6, Sesto Fiorentino, 50019 Florence, Italy; 2 Department of Chemistry, University of Florence, Via della Lastruccia 3, Sesto Fiorentino, 50019 Florence, Italy; 3 Institute for Organic Chemistry and Chemical Biology, Center for Biomolecular Magnetic Resonance (BMRZ), Johann Wolfgang Goethe-University Frankfurt, Max-von-Laue-Str. 7, 60438 Frankfurt, Germany; 4 Institute for Molecular Biosciences, Center for Biomolecular Magnetic Resonance (BMRZ), Johann Wolfgang Goethe-University Frankfurt, Max-von-Laue-Str. 7, 60438 Frankfurt, Germany; 5 Institute of Biophysical Chemistry, Center for Biomolecular Magnetic Resonance (BMRZ), Johann Wolfgang Goethe-University Frankfurt, Max-von-Laue-Str. 7, 60438 Frankfurt, Germany; 6 Department of Biology, Technical University of Darmstadt, Schnittspahnstr. 10, 64287 Darmstadt, Germany; 7 Department of Pharmacy, University of Patras, 26504 Patras, Greece

**Keywords:** SARS-CoV-2, Non-structural protein, Macrodomain, Solution NMR-spectroscopy, Protein drugability, COVID19-NMR

## Abstract

The SARS-CoV-2 genome encodes for approximately 30 proteins. Within the international project COVID19-NMR, we distribute the spectroscopic analysis of the viral proteins and RNA. Here, we report NMR chemical shift assignments for the protein Nsp3b, a domain of Nsp3. The 217-kDa large Nsp3 protein contains multiple structurally independent, yet functionally related domains including the viral papain-like protease and Nsp3b, a macrodomain (MD). In general, the MDs of SARS-CoV and MERS-CoV were suggested to play a key role in viral replication by modulating the immune response of the host. The MDs are structurally conserved. They most likely remove ADP-ribose, a common posttranslational modification, from protein side chains. This de-ADP ribosylating function has potentially evolved to protect the virus from the anti-viral ADP-ribosylation catalyzed by poly-ADP-ribose polymerases (PARPs), which in turn are triggered by pathogen-associated sensing of the host immune system. This renders the SARS-CoV-2 Nsp3b a highly relevant drug target in the viral replication process. We here report the near-complete NMR backbone resonance assignment (^1^H, ^13^C, ^15^N) of the putative Nsp3b MD in its apo form and in complex with ADP-ribose. Furthermore, we derive the secondary structure of Nsp3b in solution. In addition, ^15^N-relaxation data suggest an ordered, rigid core of the MD structure. These data will provide a basis for NMR investigations targeted at obtaining small-molecule inhibitors interfering with the catalytic activity of Nsp3b.

## Biological context

Severe acute respiratory syndrome coronavirus 2 (SARS-CoV-2), the cause of the pandemic that began in early 2020 and is accompanied by the respiratory disease COVID-19, is the latest member of a *Coronaviridae* clade, which also includes SARS-CoV from 2002 and the Middle east respiratory syndrome (MERS)-CoV. The severe velocity of the virus spread demands rapid action both in the development of a vaccine and in the development of potent virus inhibitors to weaken or eliminate the symptoms, which pose a major threat to the lives of elderly people worldwide.

The ~ 30 kb long positive sense single-stranded RNA genome of SARS-CoV-2 is one of the largest known virus genomes. The SARS-CoV-2 genome contains 14 putative open reading frames (ORFs). The majority of these ORFs was shown to be translated into functional viral proteins (Gordon et al. 2020). Among the highly conserved proteins of Betacoronaviruses (Yoshimoto 2020), the ORF1a/b-coded non-structural proteins (Nsps) 1–16 form the replication/transcription-complex—an incompletely understood network of viral-viral and viral-host protein–protein and RNA–protein interactions. Besides the membrane-bound Spike protein, which is important for the entry of the virus into the cell, a number of Nsps such as the two proteases Nsp5 (Mpro) and Nsp3d (PLpro), the Nsp3b ADP-ribose-phosphatase macrodomain (MD), and the Nsp7/8/12 RNA-dependent RNA polymerase complex are obvious drug targets.

Nsp3, the largest Nsp, is one of the most intriguing SARS-coronavirus proteins, consisting of a multitude of functionally related, but nevertheless independent domains (Snijder et al. 2003). The proteolytic processing of Nsp3 from the full-length ORF1-encoded polypeptide chain yields, a 1945 amino acid long multidomain protein. Starting from the N-terminus, its individual functional domains are named Nsp3a to Nsp3e followed by the ectodomain, which is embedded between two transmembrane regions, and the C-terminal CoV-Y domain. Nsp3b is a conserved ADP-ribose binding MD. In general, the MDs of SARS and MERS are implicated to play a key role in viral replication and modulate the immune response of the host. The MDs are structurally conserved and are thought to enzymatically remove ADP-ribose, a common posttranslational protein modification. The de-ADP ribosylating function of these enzymes has evolved to protect the virus against the anti-viral ADP-ribosylation catalyzed by poly-ADP-ribose polymerases (PARPs), which are activated by the innate immune system of the host upon sensing of pathogens. Therefore, the SARS-CoV-2 Nsp3b is a highly relevant drug target in the viral replication process.

The research consortium COVID19-NMR, which was founded at the end of March 2020, rapidly and publicly supports the search for antiviral drugs by enabling an NMR-based screening approach. This requires the large-scale production of all drugable proteins and RNAs of SARS-CoV-2, as well as an extensive assignment of their NMR resonances and the determination of their structures as a prerequisite for rational drug design. We provide here the near-complete backbone assignment of the SARS-CoV-2 Nsp3b MD and thereby enable its exploitation in subsequent applications, such as drug screening and interaction mapping with amino acid resolution.

## Methods and experiments

### Construct design

This study uses the SARS-CoV-2 NCBI reference genome entry NC_045512.2, identical to GenBank entry MN908947.3 (Wu, 2020, #13). The Nsp3b domain includes amino acids V207 to K376 within the full-length Nsp3 primary sequence, as reported in previous studies 10.2210/pdb6YWM/pdb. This sequence was inserted into a pET28a( +) vector, containing an N-terminal His_6_-tag and a tobacco etch virus (TEV) cleavage site. Due to the nature of the TEV cleavage site, the produced protein contained three artificial N-terminal residues (G-2, H-1, M0) preceding the native protein sequence.

## Sample preparation

Uniformly ^13^C,^15^N-labeled Nsp3b protein was expressed in E. *coli* strain T7express in M9 minimal medium containing 1 g/L ^15^NH_4_Cl (Cambridge Isotope Laboratories), 2 g/L ^13^C_6_-d-glucose (Eurisotop) and 50 μg/mL kanamycin. Protein expression was induced at an OD_600_ of 0.7 with 0.5 mM IPTG and the cells were incubated for 13 h at 18 °C and 120 rpm shaking. The cell pellet was resuspended in buffer A (25 mM Tris–HCl–pH 8.0, 150 mM NaCl, 5 mM imidazole and 10 mM 2-mercaptoethanol), containing one protease inhibitor tablet (cOmplete™, Roche, Germany). The cells were mechanically lysed with Microfluidics M-110P at 15,000 psi (pound per square inch) under cooling with ice using three lysis cycles. The lysate was clarified from cell debris at maximal centrifugation speed for 45 min using a JLA 16.250 rotor. Clarified supernatant was purified via FPLC using two HisTrap HP columns (2 × 5 mL, GE Healthcare, USA). Bound protein was washed with 4% buffer B (buffer A + 500 mM imidazole) and eluted with 100% buffer B. Protein containing fractions were pooled and subjected to TEV cleavage over night at 4 °C while dialyzing against 25 mM Tris–HCl pH 8.0, 150 mM NaCl, 10 mM 2-mercaptoethanol. TEV protease and tag were removed via a second IMAC purification. Protein containing fractions were pooled and concentrated using Amicon Ultra-4 filtration devices (regenerated cellulose 10 kDa NMWL) and purified with a Superdex 75 26/600 PG (320 mL GE Healthcare, USA) using a buffer containing 25 mM Bis–Tris pH 6.5, 150 mM NaCl, 3 mM tris-(2-carboxyethyl)-phosphin (TCEP). The holo sample was prepared as follows. A 100 mM stock solution of ADP-ribose sodium (Sigma A0752) was prepared in water. This stock solution was used to prepare the Nsp3b-ADP-ribose complex by adding a tenfold molar excess to the protein Nsp3b (650 µM).

### NMR experiments

All experiments for the backbone assignment of both apo and ADP-ribose bound Nsp3b were recorded at 298 K using an ultra-high field Avance NEO 1.2 GHz NMR spectrometer, equipped with a 3 mm TCI H/C/N CryoProbe. All spectra were acquired using standard pulse sequences (Favier and Brutscher 2011; Lescop et al. 2007; Solyom et al. 2013) and processed using the Bruker software TopSpin 4.0.6. For the assignment, a set of double and triple resonance experiments was performed. The set of NMR experiments used for sequence specific assignment is summarized in Table 1.

**Table 1:** List of experiments collected to perform the sequence specific assignment of apo-Nsp3b (A) and ADP-ribose bound Nsp3b (B). Main parameters used are reported

Experiments	Time domain data size (points)			Spectral width (ppm)			ns	Delay time (s)
	t_1_	t_2_	t_3_	F_1_	F_2_	F_3_		
A. apo-Nsp3b								
^1^H-^15^N-HSQC	256	2048		36.5 (^15^N)	16.0 (^1^H)		4	1.2
^1^H-^15^N best-TROSY	256	2048		36.5 (^15^N)	16.0 (^1^H)		4	0.2
Best-TROSY-HN(CO)CACB	112	64	3072	75.3 (^15^N)	32.2 (^15^N)	13.9 (^1^H)	40	0.25
Best-TROSY-HNCACB	112	64	3072	75.3 (^13^C)	32.2 (^15^N)	13.9 (^1^H)	40	0.25
Best-TROSY-HN(CA)CO	104	64	3072	14.7 (^13^C)	36.5 (^15^N)	13.9 (^1^H)	40	0.25
Best-TROSY-HNCO	104	64	3072	14.7 (^13^C)	36.5 (^15^N)	13.9 (^1^H)	4	0.25
^15^N R_1_	10	128	2048	10.0 (^1^H)	35.0 (^15^N)	14.0 (^1^H)	16	1.2
^15^N R_2_	10	128	2048	10.0 (^1^H)	35.0 (^15^N)	14.0 (^1^H)	16	1.2
^15^N-NOE	2	128	2048	10.0 (^1^H)	35.0 (^15^N)	14.0 (^1^H)	16	3
B. Nsp3b-ADP-ribose								
^1^H-^15^N-HSQC	256	2048		36.5 (^15^N)	16.0 (^1^H)		4	1.2
^1^H-^15^N best-TROSY	256	2048		36.5 (^15^N)	16.0 (^1^H)		4	0.2
Best-TROSY-HN(CO)CACB	112	64	3072	75.3 (^15^N)	32.2 (^15^N)	13.9 (^1^H)	48	0.25
Best-TROSY-HNCACB	112	64	3072	75.3 (^13^C)	32.2 (^15^N)	13.9 (^1^H)	40	0.25
Best-TROSY-HNCO	104	64	3072	14.7 (^13^C)	36.5 (^15^N)	13.9 (^1^H)	4	0.25
^15^N R_1_	10	128	2048	10.0 (^1^H)	35.0 (^15^N)	14.0 (^1^H)	16	1.2
^15^N R_2_	10	128	2048	10.0 (^1^H)	35.0 (^15^N)	14.0 (^1^H)	16	1.2
^15^N-NOE	2	128	2048	10.0 (^1^H)	35.0 (^15^N)	14.0 (^1^H)	16	3

Relaxation experiments (^15^N *T*_1_, *T*_2_ and {^1^H}–^15^N NOE) were conducted on a Bruker Avance III four-channel 700 MHz NMR spectrometer equipped with a cryogenically cooled 5 mm ^1^H/^13^C/^15^N/D Z-gradient probe (TCI), at 298 K using the TROSY pseudo3D pulse sequences (Zhu et al. 2000). The delays for the ^15^N *T*1 were 20, 60, 100, 200, 400, 600, 800 and 1200 ms, while delays of 15.68, 31.36, 62.72, 94.08, 125.44, 156.80, 188.16 and 219.52 ms were used in the ^15^N *T*2 experiments. The model free approach in the Dynamic Center/Topsin3.6 software was used for data analysis and in order to obtain the *S*^2^ values. Data were fitted using a global isotropic model (M1 included in Dynamic Center software, using the equation: $j(\omega ) = (2/5)\tau_{c} \left[ {S^{2} /(1 + (\tau_{c} \omega )^{2} )} \right]$.

Proton resonances were calibrated with respect to the signal of 2,2-dimethylsilapentane-5-sulfonic acid (DSS). Nitrogen and carbon chemical shifts were referenced indirectly to the ^1^H standard using a conversion factor derived from the ratio of NMR frequencies (Wishart et al. 1995).

## Assignments and data deposition

**Fig. 1:**
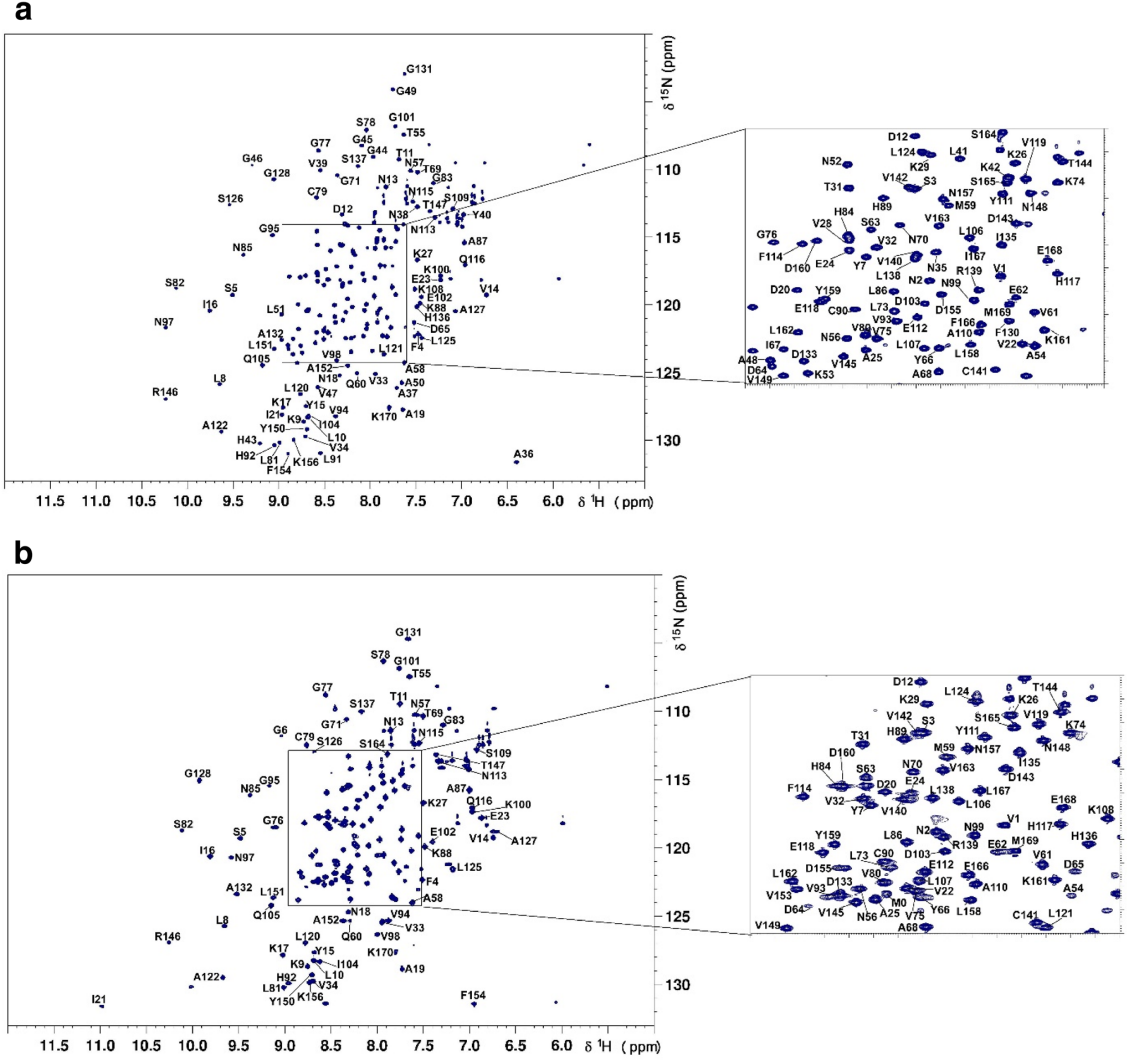
^1^H,^15^N-HSQC spectrum of the apo (**a**) and ADP-ribose bound (**b**) forms ^13^C,^15^N-labelled SARS-CoV-2 Nsp3b at 650 μM concentration in 25 mM Bis–Tris pH 6.5, 150 mM NaCl, 3 mM TCEP and 5% D_2_O measured at 298 K on a 1.2 GHz Spectrometer with chemical shift assignment depicted. Backbone NH peaks are labelled with their assignments

**Fig. 2:**
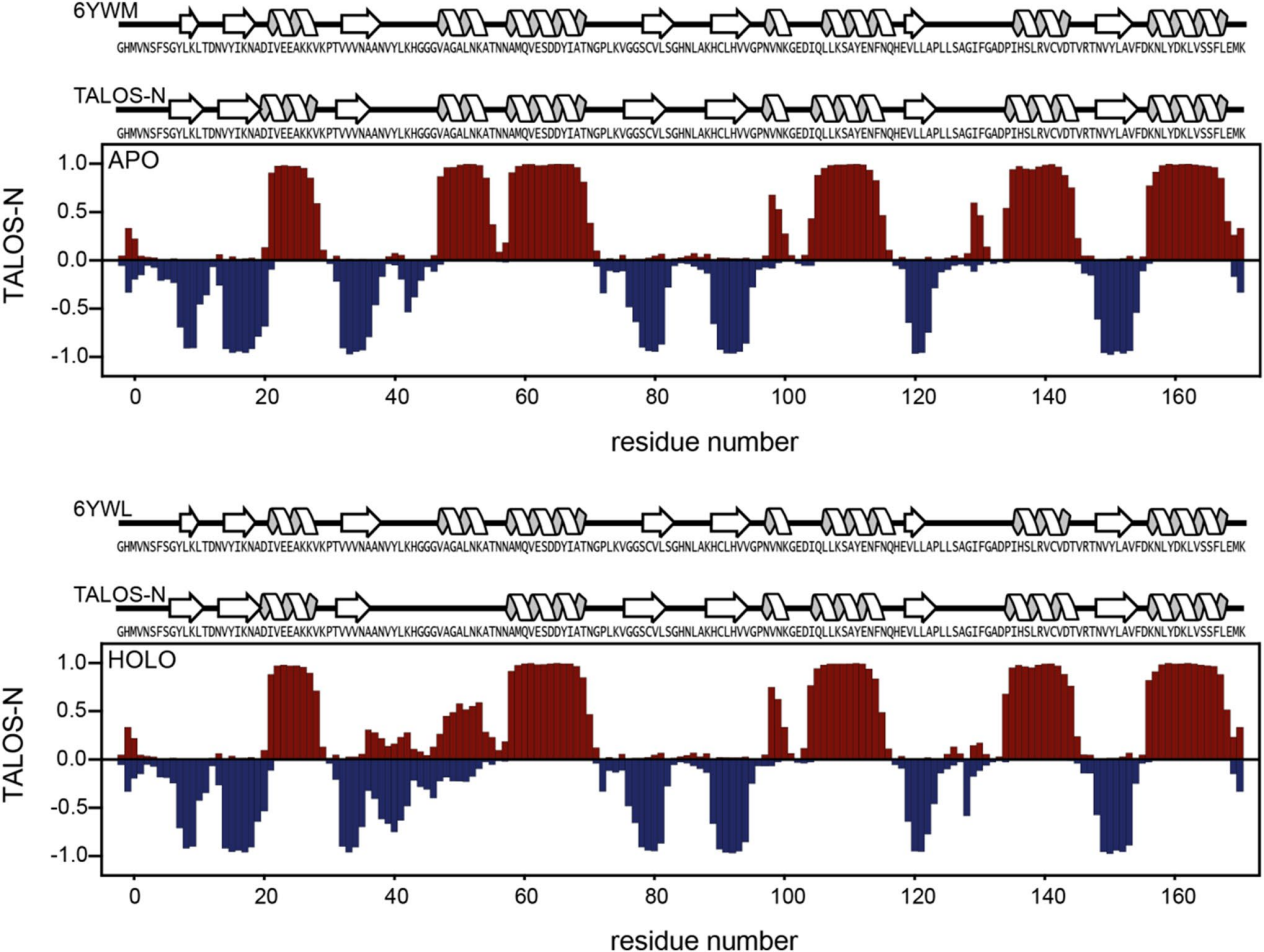
Display of TALOS predicted secondary structure for the apo (**a**) and holo (**b**) Nsp3b. For comparison secondary structure elements obtained from X-ray structures and TALOS-N (Shen and Bax 2013) are displayed on the top of each plot. For the for residues between 35 and 53 in the ADP-bound Nsp3b, the predictions were sequence based. In case of X-ray structures, the secondary structures were extracted with pdbsum (Laskowski et al. 1997) using the pdb entries 6YWM (apo) and 6YWL (ADP-ribose bound)

The ^1^H,^15^N-HSQC spectra showed well-dispersed amide signals for both apo (Fig. 1a) and ADP-ribose bound Nsp3b (Fig. 1b). Assignments of apo and ADP-ribose bound Nsp3b were performed with the program CARA (https://www.nmr.ch). For apo Nsp3b we assigned 98% of ^1^H/^15^N backbone pairs and 98.8, 99.4 and 99.4% of all CO, Cα and Cβ chemical shifts, respectively. In the case of ADP-ribose Nsp3b we assigned 86% of ^1^H/^15^N backbone pairs and 83.2, 87.3 and 89.3% of all CO, Cα and Cβ chemical shifts, respectively. The unassigned residues of the ADP-ribose bound Nsp3b correspond to the stretches Asn35-Lys53 and Ile129-Phe130 (see Fig. 2). Furthermore, none of the unassigned peaks are seen in the HSQC suggesting exchange broadening of these residues in the presence of ADP-ribose.

Secondary structure evaluation was performed using chemical shift assignments of five atoms (H^N^, Cα, Cβ, CO, N) for a given residue in the sequence with TALOS-N (Shen and Bax 2013). The results for Nsp3b and ADP-ribose bound Nsp3b (Fig. 2) are in good agreement with each other. Furthermore, we observed that the dihedral angles predicted by TALOS-N (Shen and Bax 2013) for both apo and holo Nsp3b are in excellent agreement with the dihedral angles found in the apo (6YWM) and holo (6YWL) crystal structures, indicating that ligand binding does not alter the overall the secondary structure within the MD.

**Fig. 3:**
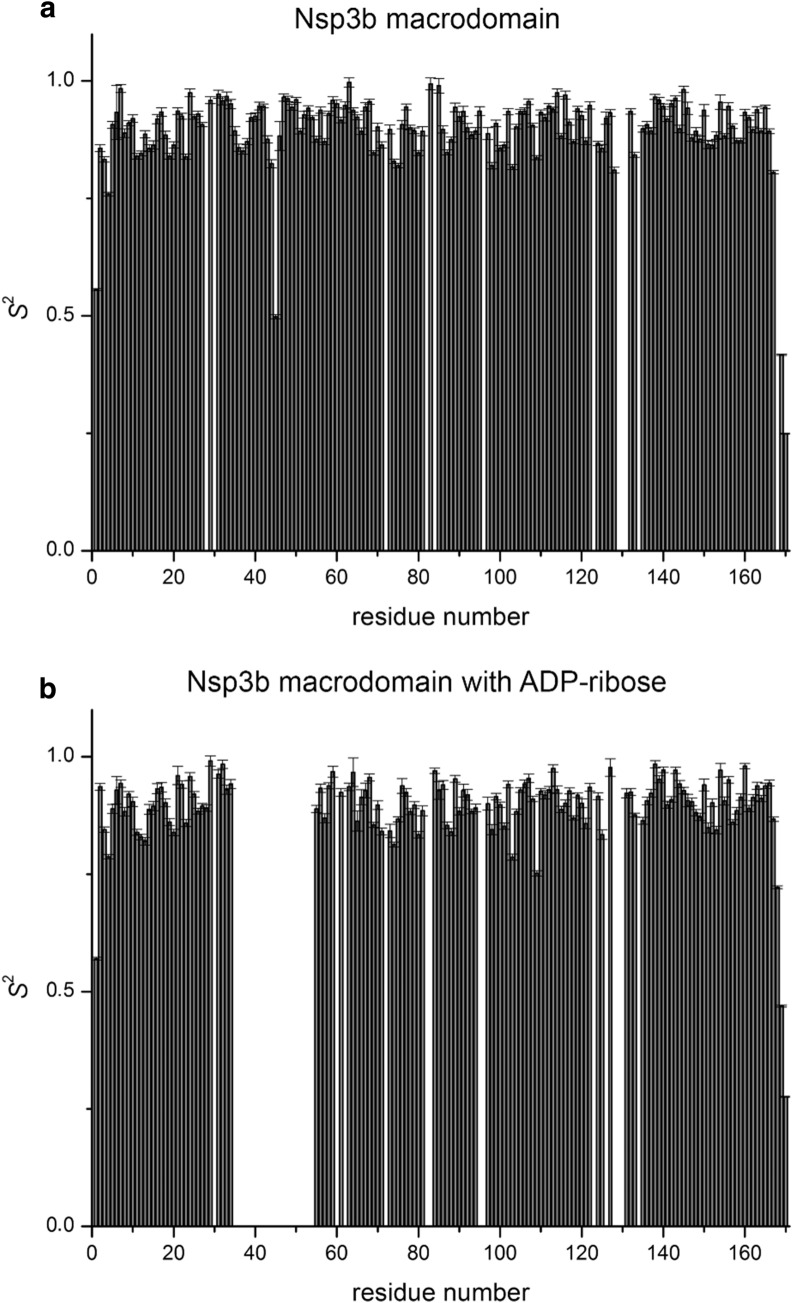
*S*^2^ order parameters of the backbone of SARS-CoV-2 Nsp3b in its apo form (**a**) and in complex with ADP-ribose (**b**). Values close to 1 suggest ordered structure on the ps/ns timescale. Errors were derived through Monte Carlo error analysis embedded in the fitting routine of Bruker software TopSpin3.6 Dynamic Center

Backbone amide order parameters *S*^2^ are presented in Fig. 3 and reveal an ordered, rigid core of the structure, with slightly flexible termini both, for the apo Nsp3b and its complexed form with ADP-ribose. The correlation time for isotropic tumbling in solution as calculated from the *R*_2_/*R*_1_ ratio is 9.15 ± 0.5 and 9.10 ± 0.5 ns for the apo Nsp3b and the holo Nsp3b-ADP-ribose complex, respectively (theoretical MW 18.65 kDa).

**Fig. 4:**
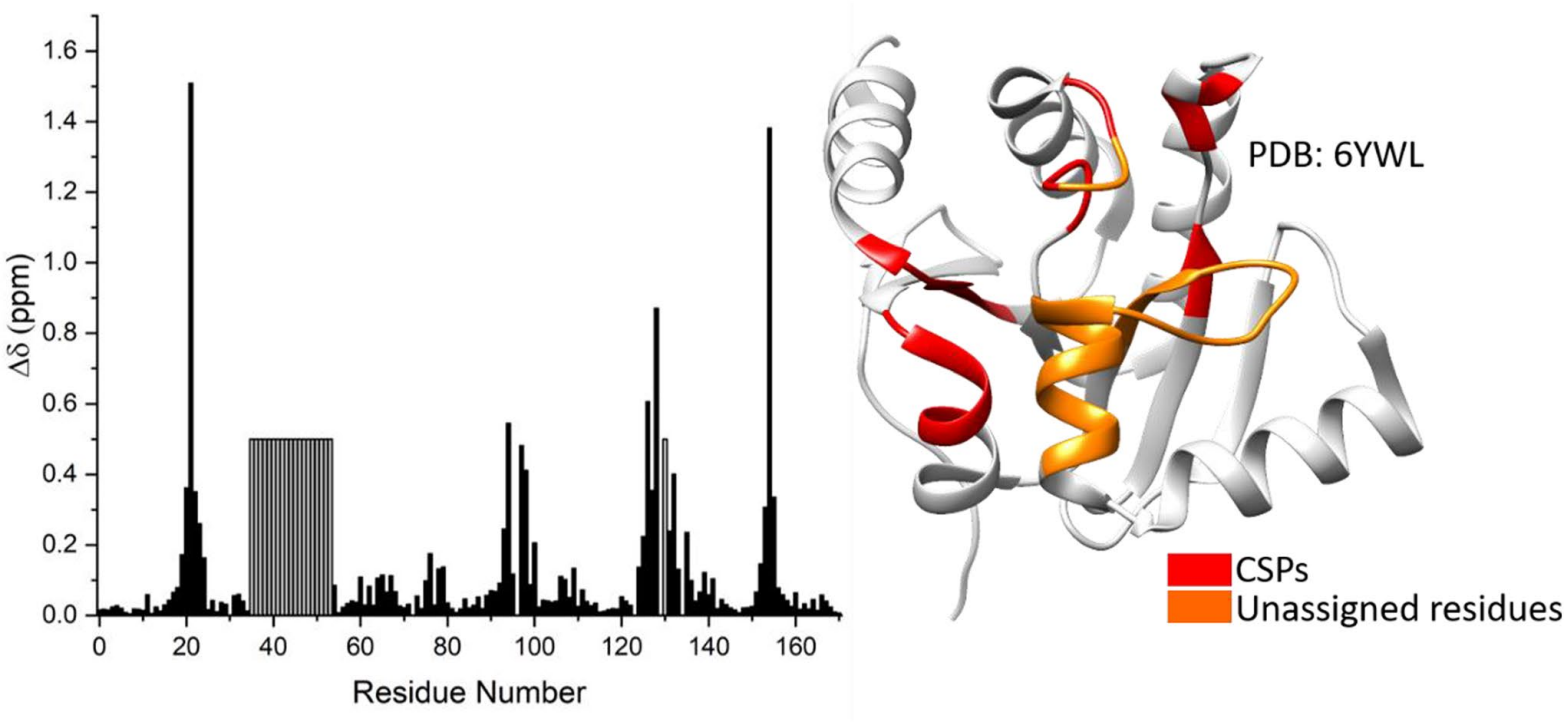
Chemical shift perturbations (CSPs) between the apo and holo Nsp3b-ADP-ribose complex are plotted as a function of Nsp3b residue number. The observed CSPs are mapped onto the crystal structure (6YWL)

ADP-ribose binds to Nsp3b with a dissociation constant (K_D_) of 13 µM (Frick et al. 2020). The position of the ADP-ribose molecule within the SARS-CoV-2 Nsp3b binding site was defined through an NMR characterization in solution (Fig. 4). The mapped binding site is in good agreement with the binding pocket observed in the crystal structure (6YWL). The chemical shift values for the ^1^H, ^13^C and ^15^N resonances of apo and holo forms of SARS-CoV-2 Nsp3b have been deposited at the BioMagResBank (https://www.bmrb.wisc.edu) under accession numbers 50387 and 50,388, respectively.
